# Assessing the feasibility of using salivary microRNAs as biomarkers to distinguish between chronic stress and childhood trauma in African American young women in an exploratory pilot study

**DOI:** 10.3389/fpsyt.2025.1507064

**Published:** 2025-02-14

**Authors:** Erica Holliday, Anisah Bagasra, Omar Bagasra, Pratima Pandey

**Affiliations:** ^1^ Department of Psychological Science, Kennesaw State University, Kennesaw, GA, United States; ^2^ Department of Biology, Claflin University, Orangeburg, SC, United States

**Keywords:** microRNA, adverse childhood experiences, social readjustment rating scale, women, *miR-19b*, *miR-187*, *miR-34a* and *miR-135-3p*

## Abstract

**Introduction:**

The current study assessed the impact of self-reported stress measures on microRNA (miRNA) profiles in saliva exosomes. Saliva is one of the most accessible and non-invasive bodily fluids and exosomal miRNAs in saliva could be useful in (1) measuring stress states and (2) distinguishing between individuals suffering from high levels of chronic stress vs. adverse childhood experiences (ACEs). miRNAs are small, noncoding RNAs that act as gene regulators. Several studies have shown differential expressions of certain miRNA in neurological diseases and in stress, post-traumatic stress syndrome (PTSD) and anxiety. Detailed analyses of miRNA expressions and profiling of miRNAs among populations with various exposures to traumatic and life stressors have not been carried out. The goal of our study was to discover miRNAs associated with high chronic stress or childhood trauma.

**Method:**

This study sought to explore miRNA expression in African American young women from a small, southern Historically Black College and University (HBCU). Twelve participants completed the social readjustment rating scale (SRRS), ACEs scale, and saliva collection and were divided into three groups based on ACE and chronic stress score: Low Chronic Stress (LCS; n = 4); High Chronic Stress (HCS; n = 4); High Chronic Stress + High ACEs (HCS+HA; n=4). A custom-made miRNA Taqman-Array tested for fold change in four miRNAs (i.e., *miR-19b, miR-187, miR-34a* and *miR-135-3p*).

**Results:**

There was a significant downregulation of miR-19b (*χ* ^2^(2, N=12) = 7.42, p < 0.01, η²= 0.915), miR-187 (*χ* ^2^ (2, N = 12) = 7.36, p < 0.05, η²= 0.598), and miR-34a (*χ* ^2^(2, N = 12) = 7.42, p < 0.05, η²= 0.60). in both the HCS and the HCS+HA groups vs. LCS. Interestingly, miR-135-3p (*χ* ^2^(2, N = 12) = 8.00, p < 0.05, η²= 0.67. was upregulated in the HCS group vs. LCS and HCS+LA. Expression for miR-135-3p was not significantly different between LCS + HCS+HA.

**Conclusion:**

Our analyses shows that miRNA extracted from salivary exosomes can be a reliable biomarker for stress and *miR-135a-3p* appears to be the most upregulated between LCS and HCS individuals and a potential candidate to corroborate self-reports on self-assessments and predict negative health outcomes. Given that HCS+HA did not show an upregulation of miR-135-3p but had similar expression in the other three miRs compared to HCS group may indicate an adaptive stress response following early life adversity. Further, downregulation in miR-135-3p in individuals with high levels of chronic stress could point to unknown childhood trauma exposure (e.g. closed adoptions, dissociative amnesia, abuse). A major limitation in this study is the small sample size and future directions include determining the predictive validity of these miRNAs in predicting onset of physical and mental health outcomes for early interventions in larger studies.

## Introduction

MicroRNAs (miR) have emerged as the critical regulators of gene expression at the post-transcriptional levels and are dynamically regulated during fear conditioning (1), stress adaptations (2, 3) and anxiety disorders (4). MiRNAs are small (18-22 nucleotides), non-coding RNAs that can regulate gene expression at transcriptional and post-transcriptional levels. Emerging data suggests human saliva may be a viable, non-invasive source to examine change in miRNA expression due to stress (5, 6) as well as detection and diagnosis of health outcomes (3, 7–13). Therefore, it would be logical to explore the role of chronic stress and stress associated with adverse childhood experiences (ACE) in regulating the expression of miRNAs to develop future non-invasive screens that predict negative health outcomes because of stress. ACEs include forms of abuse, neglect, household dysfunction, and other traumatic stressors experienced from birth to the age of 18. Kalmakis & Chandler (2015) (14) offer a more formal definition of adverse childhood experiences as “childhood events, varying in severity and often chronic, occurring in a child’s family or social environment that cause harm or distress, thereby disrupting the child’s physical or psychological health and development.” These are commonly measured using a self-report measure that gives each an ACE score based on their reported experiences.

Numerous studies have found a correlation between ACE scores and physical and mental health outcomes in adulthood. ACEs can evolve into abnormal fear processing and may result in deficiency in fear-inhibitory mechanisms and impairment in the ability to discriminate between safety and danger cues (15). Previous research on ACE indicates relationships between high ACE scores and adult alcohol problems (16), prescription drug use (17), migraines and vascular biomarkers (18), and decreased oxytocin levels (19). Higher ACE scores were correlated with anxiety disorder, PTSD, and bipolar disorder in a sample of low-income women (20). Salinas-Miranda et al., also found ACE to be linked to adult quality of life, particularly stress and sleep disorder (2015) (13). Numerous epidemiological studies suggest neurobiological impacts, particularly to the stress-response systems, from high level of ACEs (21). Early research on ACEs was conducted primarily among White, middle class populations. More contemporary research has expanded to include greater racial, ethnic, and socioeconomic diversity to explore how ACE impacts health outcomes. Studies of African Americans have found that high ACE scores are associated with greater risk of future cardiometabolic disorders and shortened leukocyte telomere length (22), and higher rates of substance use (23). Together, these studies provide evidence that early life adversity causes a wide range of negative outcomes in adulthood highlighting the need to identify biomarkers to screen for in early adulthood to lessen severity of negative outcomes later in adulthood.

Similarly, stressful life events have been found to correlate with both physical and mental health issues suggesting a greater need for understanding the role of stress interactions in onset of health issues (24, 25). Stressful life events are reported as more frequent among racial and ethnic minority groups (26) leading to a greater need for stress epigenetic research among these groups. Health-related stress events have also been linked to higher mortality rate (27) indicating the need for identifying non-invasive and high-throughput biomarker screens utilizing miRNAs found in salivary exosomes.

Several biomarkers of stress induced pathology have been evaluated, such as cortisol, salivary alpha-amylase, and inflammatory cytokines, but may be more unreliable in predicting outcomes due to modulations by circadian rhythm and other co-factors (28). miRNAs can be in various bodily fluids including blood, serum, plasma, and saliva (29). However, except for saliva, other means of exploring miRNAs are invasive. In saliva, miRNAs are well protected in exosomes and are considered a non-invasive source of biomarkers of clinically relevant consequences of stress (30). Even though miRNAs play an important role in responding to stress and other changes in a person’s environment, few miRNA studies have been conducted to understand alterations in miRNA in populations under stress (31–33) or to identify specific miRNA expressions in minority populations (34). We hypothesized that high levels of chronic stress would lead to changes in salivary microRNA expression that were distinct from low levels of chronic stress and that high levels of early life adversity, measured by the adverse childhood experience (ACE) scale would also show distinct miRNA profiles. Finally, studies focusing on African American populations, and specifically African American emerging adults are lacking in the current literature. Thus, this paper serves as a first to identify dynamic regulation of candidate miRNAs in a mostly African-American population characterized by high stress or low stress conditions that could serve as a clinically relevant biomarker screen for stress-related illnesses.

## Materials and methods

For this study we recruited female African Americans from within the university community located in a rural town (See **Table 1** for Participant Demographics). The university is a Historically Black college (HBCU) with a 95% African American student population. The majority of students are from rural areas of the Southeast United States and 85% of the student population are Pell Grant eligible, indicating families fall below the Federal Poverty Line. The student population represents young adult African Americans who are likely to have been exposed to childhood or chronic stressors related to financial hardship. Twenty-six participants completed the consent process and survey packets. Out of 26 initial participants a total of 12 participants completed the survey packets and saliva collection (morning, afternoon, and evening) to allow for miRNA analysis for this pilot study.

**Table 1:** Study sample characteristics.

Study Sample Characteristics	n	%	M	SEM
Age	12		19.73	.25
Gender				
Male	0	0%		
Female	12	100%		
Non-binary	0	0%		
Marital Status				
Married	0	0		
Single	12	100%		
Race/Ethnicity				
Black/African American	12	100%		
Alcohol Use				
Yes	2	16.6%		
No	10	83.3%		
ACE Score	12		2.58	.72
SRSS Score			368.66	51.45

Table shows demographics for participants enrolled in the study that met inclusion criteria. Adverse childhood experiences and Social Readjustment Scores are dispayed as study totals and group totals. M, means and SE, standard error of the mean.

Participants who responded to recruitment efforts on campus and met the study criteria came to the university campus, completed a consent form, and completed three questionnaires. After determining their stress using the ACE scale and the Social Readjustment Rating Scale (SRRS) they were divided into three groups: Low Chronic Stress (LCS), High Chronic Stress (HCS), High Chronic Stress + High ACEs (HCS+HE). Saliva collection took place over one week where participants signed up for a day to complete saliva collection in the morning, afternoon, and evening. Individuals who successfully submitted for saliva collection three times in one day and whose samples were viable were then analyzed.

The self-administered questionnaires consisted of 1) A demographic survey including age, gender, annual income, and health behavior survey measuring alcohol and tobacco use. The second self-administered questionnaire was the Adverse Childhood Experiences survey. This scale measures the number of Adverse Childhood Experiences before the age of 18. It consists of ten items. The lowest score a participant can earn is 0 and the highest score is 10. For the purpose of this study, scores of 3 or above were considered high scores, indicating childhood exposure to some level of abuse, neglect, or household dysfunction. The third scale was the Holmes and Rahe Social Readjustment Rating scale, which measures stressful life experience in the past year. The Holmes and Rahe Social Readjustment Rating Scale (SRSS) is a scale measuring stressful life events that have occurred in the past year (35). Each item on the scale is assigned a life change unit. Death of a spouse, partner, or parent, for example, is the life event with the highest number of life change units at 100. Minor violation of the law is a life event with the lowest number of life change units at 10. The 43 items list contains both positive and negative life events that are common stressors. The scale can be used to measure the number of life events experienced each year or by adding up the total number of life change units. The SRSS was originally developed to measure stressful life events and susceptibility to illness. Individuals scoring about 300 were found to be at greater risk of illness or health change in the coming year (35). For the purpose of this study, scores of 300 or more were therefore considered the cut off for high chronic stress scores. Studies using the SRSS have consistently found a relationship between psychological distress and subsequent occurrence of illness, despite criticisms of the life change unit assignment per life event.

Each participant’s survey packet and saliva samples were coded with a unique identifier to allow for matching a person’s miRNA profile analysis to their scores on the ACE and SRRS.

### Brief methodology

To evaluate the differentially expressed miRNAs we collected saliva samples from each participant at three different occasions during a single day. The exosomal miRNAs from each of the individual samples were isolated by utilizing total saliva exosome miRNA kit (Invitrogen Cat # 4478454) and characterized miRNA profile by utilizing custom made human miRNA proofing kit (ThermoFisher) Differentially expressed miRNAs were identified and statistically significant changes in miRNA expression were correlated with ACE and SRRS scores (**Table 2**).

**Table 2:** SRSS and ACE Scores for Participants.

Descriptives - SRSS					
Group	N	Mean	SD	SE	Coefficient of variation
Low Stress	4	153.250	70.938	35.469	0.463
High Stress	4	448.750	99.697	49.849	0.222
High Stress + High ACE	4	504.000	81.862	40.931	0.162
Descriptives - ACEs					
Group	N	Mean	SD	SE	Coefficient of variation
Low Stress	4	0.000	0.000	0.000	NaN
High Stress	4	2.250	0.957	0.479	0.426
High Stress + High ACE	4	5.500	1.291	0.645	0.235

Table shows means and variances for the Social Readjustment Rating Scale (SRSS) and Adverse Childhood Experiences for each group. SD, standard deviation; SE, Standard Error.

### Saliva collection

Saliva samples were collected three times from each of the individuals: first, in the morning (7:30 AM to 10:00 AM). In the afternoon (12:00 PM to 2:30 PM), and in the evening 5 PM to 7 PM). These times of collections were chosen to normalize the biological changes that occur during the circadian rhythm. During the collection period, participants were seated straight up and were instructed to refrain from speaking or swallowing. They allowed the saliva to accumulate in the floor of the mouth, and then spit it through a funnel into a pre-cooled saliva tube. Saliva was collected using the Saliva RNA Collection and Preservation Device (Norgen, Cat#RU53810). At least 1 mL of whole saliva was collected. Salivary flow rate was calculated by dividing the volume of collected saliva by the duration of collection time. After collection of saliva, the specimens were stored at 4°C for up to 6 h, after which it was stored at −80°C until use. The miRNAs were isolated the same day of collection by utilizing exosome mRNA collection kit (Invitrogen).

### Salivary exosome isolation

Exosomes were isolated from saliva samples (0.5–1.0 mL) using total exosome isolation reagent (Invitrogen, Carlsbad, CA, USA), in accordance with the manufacturer’s protocol and using polyethylene glycol (PEG) based reagents for exosome precipitation (36). Briefly, whole saliva samples were centrifuged at 2000× *g (gravity)* for 10 **min** at room temperature to remove cells and debris. Supernatant were incubated with total exosome isolation reagent (Invitrogen Cat# 4484453) at 8°C for 1 **h**. After incubation, samples were centrifuged at 10,000× *g* for 1 **h** at 8°C. Residual supernatants were then discarded, and the exosome pellets were collected. Following exosome isolation, the pellet was treated with Rnase A to degrade any residual cellular RNAs in order to ensure that all detected RNA was exosomal in origin. The pellets were then re-suspended in a 200 μL volume (calculated by salivary supernatant volume) of exosome re-suspension buffer (provided by the manufacturer-Invitrogen).

### MiRNA isolation and purification

In order to isolate and purify the miRs, we utilized TaqMan miRNA ABC purification kit (Applied Biosystems Cat #4473087). We isolated miRNAS according to the manufacturer’s protocol. Briefly, the total exosomal miRNAs isolated from each of the individual were transferred to tubes containing miRNA-beads, then the miRNAs were hybridized to the beads. The beads were isolated by magnetic rack, washed and miRNAs were eluted using 70°C Thermomixer at 1200 rpm.

### Custom RT and preamplification pools on custom TaqMan array microRNA cards

We utilized Applied Biosystem “Custom RT and Preamplification (pools on Custom TaqMan Array microRNA Cards (Life Technologies, protocol #4478705) to analyze the differential expression of nine miRNAs. The cDNA synthesis was performed using customized miRNA Reverse Transcriptase (RT) pool without amplification. The comparative RT-PCR was performed with internal control U6 for each sample, according to the manufacturers’ protocol. The RT primer pool designed to analyze saliva miRNAs consisted of 4 (*miR-19b, miR-187, miR-34a* and *miR-135-3p*) miRNA specific primers sets, respectively. The following custom primers were used:

hsa-miR-19b-3p MIMAT0000074 UGUGCAAAUCCAUGCAAAACUGA;
hsa-miR-187-3p MIMAT0000262 UCGUGUCUUGUGUUGCAGCCGG;
hsa-miR-34a-3p MIMAT0004557 CAAUCAGCAAGUAUACUGCCCU;
hsa-miR-135a-3p MIMAT0004595 UAUAGGGAUUGGAGCCGUGGCG

A positive control reverse transcription reaction with the small nucleolar RNA U6 was performed using specific primers. These miRNAs were selected based on their relevance and expression levels in human saliva and plasma with respect to stress and anxiety.

### Comparative PCR analyses

After cDNA synthesis of samples, utilizing internal control U6, comparative real time PCR was carried out. Taqman^®^ Universal Master Mix II (cat# 4440040) supplied by applied biosystem was used. Custom design plates with microRNA specific primers, Taqman^®^ probe NGF-MGB, Taqman^®^ assay 20x and passive reference ROX was used. 10 µl of cDNA sample and Taqman^®^ master mix was added to bring the total volume of 20 µl. The cycle condition consisted of polymerase activation at 95°C for 10 minutes, then 40 cycle denaturation at 95°C for 15 seconds, followed by annealing/extension at 60°C for 1 minute. The experiment was repeated three times.

For internal control U6 Taqman^®^ microRNA assay (Cat# 4427975) supplied by applied biosystem was used to perform RT-PCR. 1 µl Taqman^®^ microRNA assay 20x, 5 µl of cDNA, 10 µl of PCR master mix, 4 µl of nucleases free water was mixed to make final volume of 20 µl. Delta cycle threshold was utilized to calculate the Fold Change for each microRNA sample.

### ΔCT calculation and 2^ΔCT method

Threshold cycle (CT) values were obtained from the qPCR analysis for both the target gene and U6 (37). ΔCT was calculated for each sample by subtracting the CT value of U6 (internal control) from the CT value of the target gene:


$$\text{Δ}\text{CT}={\text{CT}}_{\text{target}}-{\text{CT}}_{\text{U}6}$$

To calculate the relative expression of the target gene normalized to U6, the following formula was applied:


$$\text{Relative Expression}=2^{-\text{Δ}\text{CT}}$$

This equation derives from the assumption that a decrease of one CT corresponds to a doubling of the gene product. Therefore, 2^−ΔCT^ provides a measure of how much more or less the target gene is expressed relative to the U6 control. A smaller ΔCT value leads to a larger 2^−ΔCT^ indicating higher gene expression, while a larger ΔCT indicates lower expression. All reactions were performed in triplicate, and mean 2^−ΔCT^ values were calculated for each sample. Data were expressed as mean ± standard deviation.

### Discrimination power of candidate miRNA biomarker for various degrees of stress

To evaluate the discrimination power of candidate miRNA biomarker for Stress in a limited sample size, we carried out a non-parametric Kruskal-Wallis test with Dunn’s *post-hoc* comparisons of four miRNAs expressions in three group of individuals: i) with low chronic stress, ii) High chronic stress and iii) high chronic stress with high ACE. Pearson correlations were run between candidate miRNA, SRSS, and ACEs. Significance was determined by *p* < 0.05. Statistical analysis was performed using JASP.

## Results

In the present study, we aim to identify miRs associated with high self-reported stress over the last year without ACE and with ACE as compared to controls with low stress over the last year in expression of salivary exosomal miRNAs. The major aim of the study was to identify certain miRNAs as a phenotypical marker of high stress conditions by characterizing miRs that are either significantly upregulated or downregulated as a function of both recent stress and ACEs.

### miR-19b

**Figure 1:**
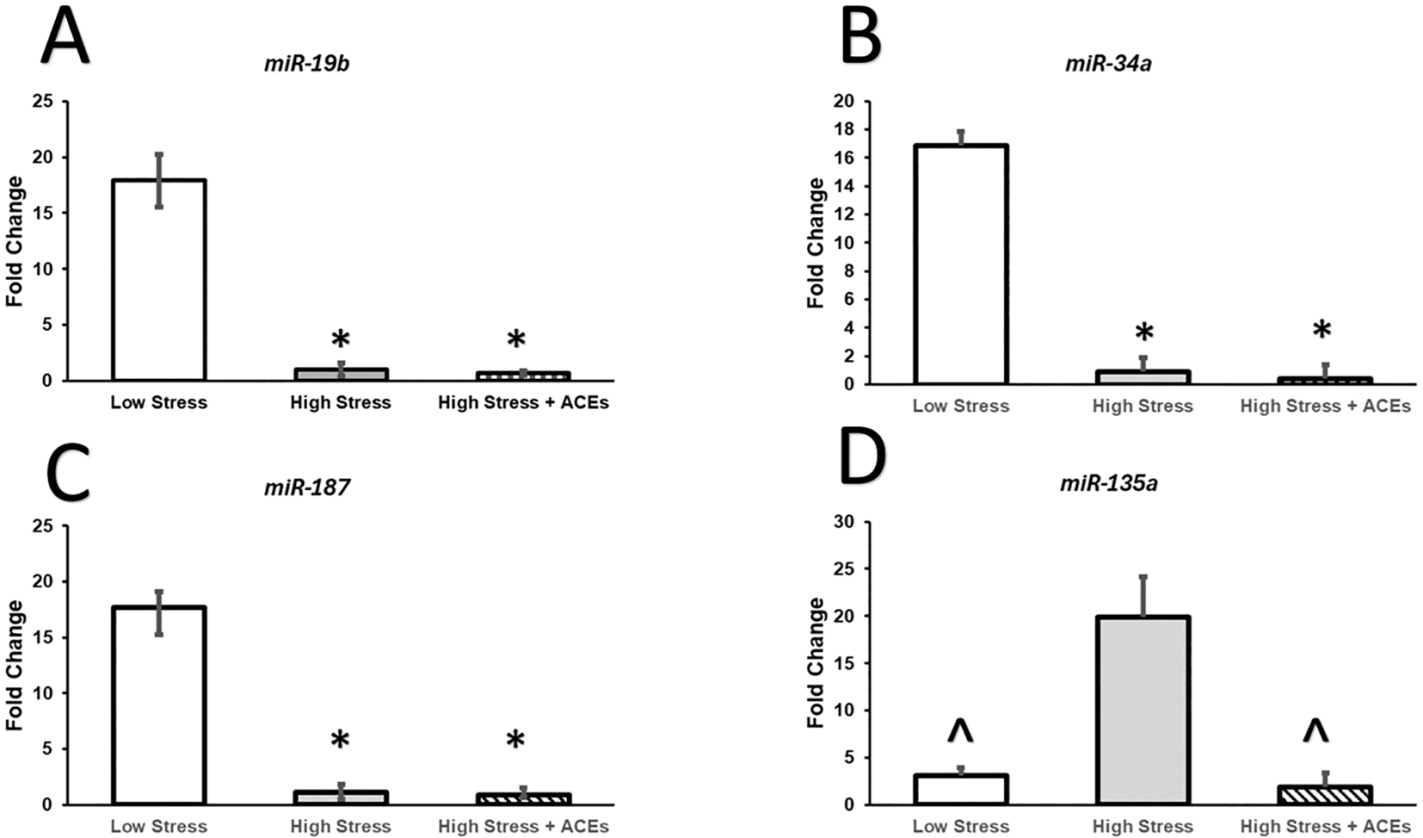
Relative expression of miRNAs is altered by High Chronic Stress and Adverse Childhood Experiences. **(A–C)** Relative expression of miR-19b, miR-34a, and miR-187 is decreased in individuals with High Chronic Stress (HCS) and High Chronic Stress coupled with High ACEs (HCS+HA). **(D)** Levels of High Chronic Stress increase expression of miR-135a compared to Low Chronic Stress (LCS) and High Chronic Stress coupled with High ACEs (HCS+HA). The decreased expression of miR-135a in High Stress with High ACEs could reflect an adaptive stress response due to the early insult to the stress response system. This profile could also help identify women with unknown childhood trauma and improve health intervention efficacy as early life stress manifests neurobiological alterations differently than chronic stress in adulthood. Bars represent group means and SEM is plotted by error bars. * depicts significant Bonferroni *post hoc* compared to LCS, ^ depicts significant Bonferroni *post hoc* compared to HCS.

Results from a Kruskal-Wallis test were significant (χ^2^(2, N=12) = 7.42, p < 0.01, η²= 0.915). Dunn’s *post-hoc* tests show LCS was significantly different compared to HCS (z=2.45, p <0.05) and HCS + HA (z=2.25, p <.05). HCS and HCS + HA were not significantly different (**Figure 1A**).

### miR-187

Results from a Kruskal-Wallis test were significant (χ^2^ (2, N = 12) = 7.36, p < 0.05, η²= 0.598. Dunn’s *post-hoc* tests show miR-187 in the LCS condition was significantly higher compared to HCS (z=2.35, p < 0.05) and HCS + HA (z = 2.35, p < 0.05). HCS and HCS + HA were not significantly different (**Figure 1B**).

### miR-34a

Results from a a Kruskal-Wallis test were significant (χ^2^(2, N = 12) = 7.42, p < 0.05, η²= 0.60. *Post-hoc* tests show miR-34a in the LCS condition was significantly higher compared to HCS (z = 2.45, p < 0.05) and HCS + HA (z = 2.26, p < 0.05). HCS and HCS + HA were not significantly different (**Figure 1C**).

### mir-135a-3p

Results from a Kruskal-Wallis test were significant (χ^2^ (2, N = 12) = 8.00, p < 0.05, η²= 0.67. *Post-hoc* tests show miR-135a-3p in the LCS condition was significantly lower compared to HCS (z=-1.96, p < 0.05) but was not different compared to HCS + HA. miR-135a-3- was significantly higher in the HCS compared to the HCS + HA condition (z = 2.75, p < 0.05) **Figure 1D**.

### Adverse childhood experiences

**Figure 2:**
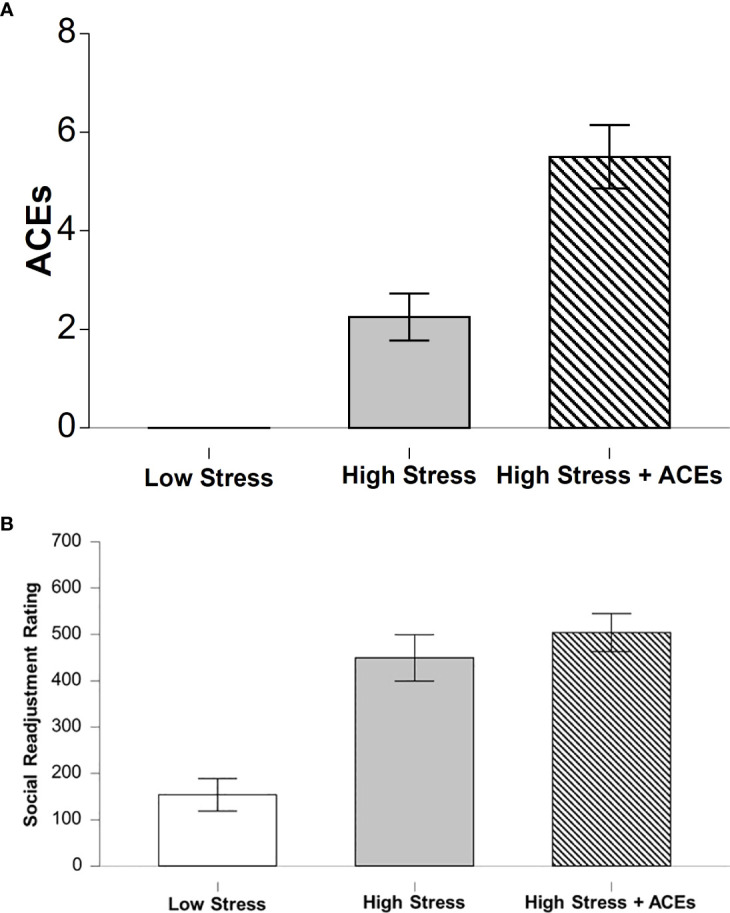
Self-reports of early life adversity and current chronic levels of stress. **(A)** The low stress group (LCS) had fewer reported ACEs compared to both stress groups and the highest incident was found in the High Stress + ACEs (HCS+HA) group. **(B)** The High Stress (HCS) and the High Stress + ACEs (HCS+HA) groups report comparable levels of high stress incidents over the last year. Bar graphs depict means and error bars reflect SEM. * depicts significant Bonferroni *post hoc* compared to LCS, ^ depicts significant Bonferroni *post hoc* compared to HCS+HA.

There was a significant main effect between groups on number of ACEs reported (χ^2^ (2, N = 12) = 10.24, p < 0.01). LCS had significantly lower reported ACEs compared to HCS + HA (t(11) = -8.38, p < 0.001) (**Figure 2A**). There were no significant differences between LCS and HCS.

### Social readjustment rating scale

There was a significant difference between groups on self-reported stressful events rated by the SRSS (χ^2^(2, N = 12) 7.54, p < 0.05, η²=.62 LCS had significantly lower SRSS scores compared to HCS (z = -2.16, p < 0.05) and HCS + HA (z = -2.55, p < 0.01). There was not a significant difference between HCS and HCS + HA **Figure 2B**. Further, there was a significant positive correlation between ACEs and SRSS (r = .82, p < 0.001).

## Discussion

It is well documented that childhood adversity and chronic stress increase risk factors for many ailments and diseases including cardiovascular disease, autoimmune disorders, cancer and increased risk for addiction, culminating in premature death. Thus, it is vital to develop reliable, objective, non-invasive screens to monitor and prevent these consequences. Exosomal miRNAs extracted from saliva are a great candidate due to the non-invasive and relative ease of collection methods. miRNAs have become the focus of various studies given their role in the regulation of the central nervous system (CNS) through post-transcriptional gene silencing (38, 39). miRNAs are known to be important mediators of the brain genomic response to stress, including the hypothalamic-pituitary-adrenal axis and the autonomic nervous system (40, 41). Results from the current study demonstrate downregulation of candidate miRNAs as well as demonstrating a possible phenotype for stress adaptation due to cumulative effects of early life adversity coupled with chronic stress. These results also provide evidence that the subjectiveness of stressful experiences correlate with objective changes in epigenetic markers of stress.

Our results support existing literature demonstrating the causal roles of *miR-19b, miR-187, miR-34a* and *miR-135-3p in* regulating stress response. For example, decreased expression of miR-19b following trauma is positively associated with onset of PTSD in women but not men (42). Results from our current exploratory study corroborate this finding and is especially relevant given the participants were exclusively African American females, similar to the characteristics of study participants from Linnstaedt et al. Cortisol exposure also downregulated miR-19b expression in rat hippocampal progenitor cells (43) and future studies aim to examine how decreased miR-19b expression impacts learning and memory or increase risk for psychological disorders.

Both the HCS and the HCS + HA displayed decreased expression of miR-187. This is alarming given the role miR-187 in suppressing tumor growth (44, 45), suggesting a possible mechanism for chronic stress increasing cancer risk. In addition to increased cancer risk and poorer disease progression prognosis associated with decreased miR-187 expression, Zhang et al. (2018) (41) found lower expression of miR-187 induces retinal cell apoptosis. Further, patients with coronary artery disease also present with a significant decrease in miR-187 expression and experimental overexpression through knockdown of its downstream target, DYRK2, improved cardiomyocyte apoptosis in a hypoxia/reoxygenation model of myocardial infarction (46). Together with the results of this study findings indicate screens targeting miR-187 in early adulthood could aid in mitigating the risk of cancer, progressive vision loss, and cardiovascular disease in at-risk demographics, such as individuals exposed to early life adversity and high levels of chronic stress. Future work should examine if health interventions can increase the expression of miR-187 to improve health outcomes.

Similar to mi-187, both HCS and HCS + HA displayed decreased expression in salivary exosomes and downregulation of mir-34a is found in breast cancer, colon cancer, prostate cancer, and vascular disease (47). Others have noted appropriate levels of miR-134a act as a critical controller of tumor suppression such that overexpression of miR-134a is currently being investigated in clinical trials for cancer treatment (48). Recent studies show patients diagnosed with major depression disorder (MDD) demonstrate downregulation of plasma miR-134a and can be used to discriminate between controls, bipolar disorder, and schizophrenia (49). Further, early life stress and chronic levels of stress increase the risk for MDD (50, 51). Taken together with our results showing both high chronic stress and high rates of ACEs downregulate saliva miR-187 expression supports the idea of using miR-187 extracted from salivary exosomes as a non-invasive screen for predicting negative health outcomes.

High chronic stress levels increased expression of miR-135-3p compared to low chronic stress group and high chronic stress plus high ACEs group. An intriguing finding that supports others’ findings early life stress programs a maladaptive stress response stress experiences in adulthood (52, 53). For example, chronic stress exposure during adulthood increases both basal cortisol levels and cortisol responses to acute stressors (54, 55), but early life adversity blunts the cortisol response to acute stressors (56). Low levels of cortisol are observed in patients diagnosed with PTSD resulting from adverse early life abuse (57). Future work should further investigate disparate health outcomes in individuals with chronic stress exposure in adulthood vs. early life stress as well as the cumulative effects. The decrease in miR-135-3p expression observed in high chronic stress + HA compared to high levels of chronic stress could serve as a useful tool to parse out individuals with undisclosed or unknown early life stress, such as in cases of adoption or parental estrangement, to better screen for associated diseases and psychological disorders in these individuals and positively benefit public health initiatives.

### Limitations

Stress and stress related disorders are among the most prevalent psychiatric disorders and are the leading cause of global morbidities. In our preliminary pilot study we have identified dysregulations in four miRNAs that have a potential to be important biomarkers in salivary exosomes and be used to target interventions that increase stress resiliency and reduce the risk and severity of psychiatric disorders. Despite our small sample size in this exploratory pilot study, we were able to achieve large effect sizes in our statistical tests. However, the homogeneity and small sample of study participants limits generalizability. Additionally, we did not have enough participants who fell into a low stress/high ACE category to analyze this group. Next steps include replicating in a larger, heterogenous sample. In our current study we have not addressed the genes or mRNAs that are associated with downregulations or upregulations of human miRNAs as this was outside the scope of this feasibility study. There are 30-40 downstream targets of any one miRNAs and future preclinical and clinical work can identify downstream targets that could be screened for simultaneously to increase predictive validity.

## Data Availability

The original contributions presented in the study are included in the article/supplementary material, further inquiries can be directed to the corresponding author/s.
